# Real-Time Telemetry System for Amperometric and Potentiometric Electrochemical Sensors

**DOI:** 10.3390/s110908593

**Published:** 2011-09-02

**Authors:** Wei-Song Wang, Hong-Yi Huang, Shu-Chun Chen, Kuo-Chuan Ho, Chia-Yu Lin, Tse-Chuan Chou, Chih-Hsien Hu, Wen-Fong Wang, Cheng-Feng Wu, Ching-Hsing Luo

**Affiliations:** 1 Department of Electrical Engineering, National Cheng Kung University, Tainan 70101, Taiwan; E-Mails: n2895145@ccmail.ncku.edu.tw (W.-S.W.); bookteam@hotmail.com (S.-C.C.); 2 Graduate Institute of Electrical Engineering, National Taipei University, Taipei 10617, Taiwan; E-Mail: hyhuang@mail.ntpu.edu.tw (H.-Y.H.); 3 Department of Chemical Engineering, National Taiwan University, Taipei 10617, Taiwan; E-Mails: kcho@ntu.edu.tw (K.-C.H.); r92524040@ntu.edu.tw (C.-Y.L.); 4 Department of Chemical Engineering, Tatung University, Taipei 104, Taiwan; E-Mail: tcchou@ttu.edu.tw (T.-C.C.); 5 Department of Chemical Engineering, National Cheng Kung University, Tainan 70101, Taiwan; E-Mail: ilovenba@ms2.url.com.tw (C.-H.H.); 6 Department of Computer Science and Information Engineering, National Yunlin University of Science & Technology, Yunlin 64002, Taiwan; E-Mails: wwf@yuntech.edu.tw (W.-F.W.); wcf9263622@hotmail.com (C.-F.W.)

**Keywords:** electrochemical sensor, telemetry system, potentiostat, instrumentation amplifier

## Abstract

A real-time telemetry system, which consists of readout circuits, an analog-to-digital converter (ADC), a microcontroller unit (MCU), a graphical user interface (GUI), and a radio frequency (RF) transceiver, is proposed for amperometric and potentiometric electrochemical sensors. By integrating the proposed system with the electrochemical sensors, analyte detection can be conveniently performed. The data is displayed in real-time on a GUI and optionally uploaded to a database via the Internet, allowing it to be accessed remotely. An MCU was implemented using a field programmable gate array (FPGA) to filter noise, transmit data, and provide control over peripheral devices to reduce power consumption, which in sleep mode is 70 mW lower than in operating mode. The readout circuits, which were implemented in the TSMC 0.18-μm CMOS process, include a potentiostat and an instrumentation amplifier (IA). The measurement results show that the proposed potentiostat has a detectable current range of 1 nA to 100 μA, and linearity with an R^2^ value of 0.99998 in each measured current range. The proposed IA has a common-mode rejection ratio (CMRR) greater than 90 dB. The proposed system was integrated with a potentiometric pH sensor and an amperometric nitrite sensor for *in vitro* experiments. The proposed system has high linearity (an R^2^ value greater than 0.99 was obtained in each experiment), a small size of 5.6 cm × 8.7 cm, high portability, and high integration.

## 1. Introduction

Electrochemical sensors are widely utilized in many applications, such as disease diagnosis, environmental monitoring, and food inspection, due to their fast analysis, high selectivity, high sensitivity, and simplicity [1]. An electrochemical sensor acts as a transducer which converts the chemical quantity of analytes within a given solution into electrical signals. In general, two forms of output signal are generated, namely potential and current, each requiring its own readout circuit. Amperometric electrochemical sensors, which generate a current signal, utilize a potentiostat as an interface circuit during the gathering of signals [2]. Potentiometric electrochemical sensors, which generate a potential signal, utilize an instrumentation amplifier (IA) as the readout circuit [3].

A back-end circuit system is necessary for acquiring sensor information and transmitting the acquired data. Consequently, the development of an electrochemical sensing telemetry system that targets clinical analytes such as nitrite and pH, both of which are essential tests for urinary tract infection (UTI), is desirable. Some research has been conducted to develop a portable system using discrete components for electrochemical sensor applications [4–6]. A telemetric potentiometric electrochemical sensor was developed to sense pH and transmit the measured signals wirelessly [7]. Additional research has been devoted to the development of a readout chip for various electrochemical sensor applications [8–10].

With the rapid development of the semiconductor industry, system-on-chip (SoC) technologies have had a substantial impact on system integration. Portable application requirements include low power consumption, compact size, and wireless communication. For portable applications and offering freedom of mobility of users, this work presents a high-integration telemetry system with readout circuits and a microcontroller unit for amperometric and potentiometric electrochemical sensor applications. The readout circuits, which include a potentiostat and an instrumentation amplifier (IA), were implemented in the TSMC 0.18-μm CMOS process. The microcontroller unit was implemented using a field programmable gate array (FPGA). The system can display acquired data in real-time on a graphical user interface (GUI) and is small enough to be portable. By integrating electrochemical sensors with the proposed system, the detection of analytes can be conveniently performed. A potentiometric pH sensor and an amperometric nitrite sensor were tested; the results verify the feasibility of the proposed system.

The rest of this article is organized as follows. Section 2 describes the proposed real-time telemetry system. In Section 3, the measurement results of the proposed system are shown. Finally, the conclusions are given in Section 4.

## 2. Proposed Real-Time Telemetry System

**Figure 1.**
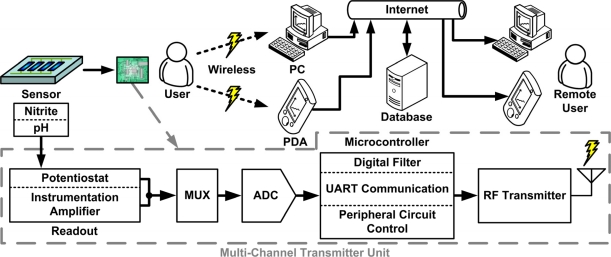
Block diagram of proposed real-time telemetry system.

The proposed telemetry system, illustrated in Figure 1, consists of front-end readout circuits, a multiplexer (MUX), an analog-to-digital converter (ADC), a microcontroller unit (MCU), a radio frequency (RF) transceiver module, a GUI, and a database. The chemical quantity of analytes is converted into electrical signals by the electrochemical sensors. The electrical signals are amplified and converted into digital form by the readout circuit and the ADC, respectively. The digital signals are processed by the MCU and transmitted to a computer or a personal digital assistant (PDA) via RF transmission for display on a GUI. The data are optionally uploaded to a database over the internet, allowing remote access. The blocks of the system are described in detail below.

### 2.1. Readout Circuits

A potentiostat and an IA are adopted as the readout circuits for amperometric and potentiometric electrochemical sensors, respectively. The cell potential (*V_cell_*), which is the potential difference between the working electrode (WE) and the reference electrode (RE) of an amperometric sensor, varies with the sensor. The maximum supplied cell potential is determined by the supply voltage of the potentiostat. The number of detectable analytes decreases with scaling down supply voltage. Therefore, to maximize the number of detectable analytes in low-voltage processes, the rail-to-rail input common-mode range (ICMR) and the rail-to-rail output swing are implemented in the readout circuits.

#### 2.1.1. Instrumentation Amplifier

**Figure 2.**
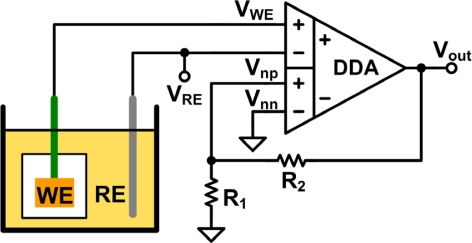
Set-up of the DDA circuit for open-circuit potential measurement.

In potentiometric experiments, the open-circuit potential is measured. A potentiometric electrochemical cell is usually composed of a WE and an RE. The open-circuit potential is the potential of the WE relative to the RE when no current flows through the cell during measurement [2]. Thus, the input impedance of the terminals, which are connected to the WE and the RE, should be as high as possible. A CMOS IA is adopted to measure the open-circuit potential. High impedance is achieved by connecting the electrodes to the gate terminals of MOSFETs. An IA also has a high common-mode rejection ratio (CMRR). The triple-opamp IA [10], a basic architecture of IA, has some drawbacks when implemented. First, mismatches of resistors and operational amplifiers decrease its CMRR. Second, the presence of three op-amps in the architecture increases complexity, which in turn increases power consumption and noise. To overcome these issues, architectures such as the current balance instrumentation amplifier (CBIA) [11], the operational transconductance amplifier (OTA) [12], and the differential difference amplifier (DDA) [13] have been proposed. However, the OTA does not have a sufficiently high input impedance for this work and its CMRR is lower than those of other architectures. The CBIA and the DDA can obtain high CMRR without requiring precisely matched resistors. However, the CBIA suffers from current mirror mismatch, which decreases its CMRR. This problem has been resolved by using a calibration technique or regulated cascode current mirrors [11]. Although the CMRR is increased, the voltage headroom may be decreased, making the CBIA unsuitable for operation with a low supply voltage. The DDA architecture is suitable for low-supply-voltage operation [13] and it maximizes the voltage headroom for multiple-sensor applications. Consequently, the DDA architecture is adopted here for measuring the open-circuit potential in potentiometric experiments. The set-up of the DDA circuit for open-circuit potential measurement is shown in Figure 2. The closed-loop gain is determined by R_1_ and R_2_. The ideal output of the negative feedback DDA circuit is expressed by:

(1) $$V_{\mathrm{out}}=\left(1+\frac{R_{2}}{R_{1}}\right)\left(V_{\mathrm{WE}}-V_{\mathrm{RE}}\right)+V_{\mathrm{nn}}$$

**Figure 3.**
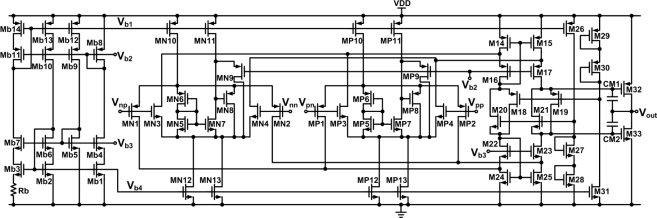
Schematic of the DDA.

A DDA with rail-to-rail ICMR and rail-to-rail output swing is implemented to provide the maximum dynamic range at the input and output, respectively, for various potentiometric sensor applications. Figure 3 shows a schematic of the DDA and its bias circuit. The transistor dimensions for the DDA of Figure 3 are listed in Table 1. The structure of the rail-to-rail input folded cascode operational amplifier with a class-AB output stage is used to achieve the rail-to-rail input and output range [14]. The input stage is modified to have two differential input ports. *V_pp_* and *V_pn_* are designated as the non-inverting input ports and *V_np_* and *V_nn_* are designated as the inverting input ports. The rail-to-rail ICMR is realized by placing an NMOS and PMOS differential input pair in parallel (MN1-MN4 and MP1-MP4). The floating class-AB control is formed by M18–M20. In practical applications, the open-loop gain of the DDA has a finite value and the small-signal transconductances of the non-inverting and inverting input ports are not equal. Thus, the output of the negative feedback of a DDA is given by [15]:

(2) $$V_{\mathrm{out}}=g_{\mathrm{mp}}r_{o}\left(V_{\mathrm{WE}}-V_{\mathrm{RE}}\right)-g_{\mathrm{mn}}r_{o}\left(V_{\mathrm{np}}-V_{\mathrm{nn}}\right)$$

where *V_np_* − *V_nn_* can be replaced by:

(3) $$V_{\mathrm{np}}-V_{\mathrm{nn}}=\frac{R_{1}}{R_{1}+R_{2}}\left(V_{\mathrm{out}}-V_{\mathrm{nn}}\right)$$

Rearranging the above equations, the non-ideal closed-loop transfer function can be expressed as:

(4) $$V_{\mathrm{out}}≅\frac{g_{\mathrm{mp}}}{g_{\mathrm{mn}}}\left(1+\frac{R_{2}}{R_{1}}\right)\left(V_{\mathrm{WE}}-V_{\mathrm{RE}}\right)+V_{\mathrm{nn}}$$

where *g_mp_* and *g_mn_* are the small-signal transconductances of the non-inverting and inverting input ports, respectively, and *r_o_* is the small-signal output resistance of the DDA. The closed-loop gain of the amplifier is directly affected by the ratio of *g_mp_* to *g_mn_*. However, the value of *g_m_* varies with the input common-mode voltage of each input port. For this reason, a constant-*g_m_* technique is needed to minimize the variation of the ratio of *g_m_* when the input ports have different common-mode voltages. An electronic zener diode is inserted between the input pairs to obtain a constant *g_m_* [16]. The electronic zener circuit is implemented by transistors M5–M9, M11, and M13. The zener voltage is determined by two complementary diode-connected transistors, M5, M6. Descriptions of the electronic zener topology can be found in [16]. This approach leads to fewer variations of the *g_m_* of the input stage and is also power efficient because no additional current path is introduced between the supply rails.

#### 2.1.2. Potentiostat

Amperometric electrochemical sensor structures can be divided into two types: two-electrode and three-electrode structures. The former comprises a WE and an RE. A lot of research has been devoted to the development of potentiostats for two-electrode amperometric sensor applications [17,18]. The latter comprises a WE, an RE, and a counter (or auxiliary) electrode (CE). This type is preferred over the two-electrode type in precisely controlling of the cell potential because the CE supplies current required for electrochemical reaction at the WE electrode to maintain the stability of the RE [19]. Thus, the proposed potentiostat is designed for three-electrode sensors.

A potentiostat typically consists of two main blocks: a control block and a current measurement block. The control block maintains the desired cell potential, which depends on the electrochemical sensor, between the WE and the RE. Normally, it can be realized using one of three approaches: a grounded working electrode [20,21], a grounded counter electrode [22], or a virtually grounded working electrode [23]. A grounded working electrode exhibits the best performance because it enhances the suppression of interference and noise [24].

In the current measurement block, the sensor current can be acquired through either the WE or the CE. For the former, a transimpedance amplifier is often adopted to transform the sensor current signal into a voltage signal [8,23]. However, this architecture can pick up additional interference such as environmental noise, which influences the output voltage of the measurement configuration due to the topology of the virtually grounded working electrode. For the latter, one approach is to insert the active components into the feedback loop of the control amplifier to measure the sensor current from the CE via current mirroring [21,24,25]. However, linearity may suffer because the channel length modulation effect of a MOSFET leads to a mismatched mirror current. Another approach is to insert a resistor into the feedback loop to transform the current signal. In [26], a difference amplifier and two voltage followers were used to amplify the voltage signal across the resistor to the desired amplitude; however, the CMRR of this architecture is lower than that of the triple-opamp IA. Inserting a resistor has been shown to be the most stable approach because there is no active component in the feedback loop.

**Figure 4.**
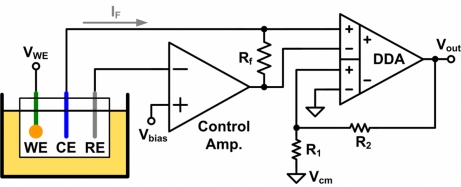
Set-up of the proposed potentiostat circuit for three-electrode amperometric sensors.

In order to linearly convert the current signal into a voltage signal without decreasing the stability of a potentiostat, a resistor is inserted into the feedback loop in the proposed architecture. The voltage signal across the resistor is then amplified by an IA, which also enhances the CMRR of the proposed potentiostat. Figure 4 shows the set-up of the proposed potentiostat circuit for three-electrode amperometric sensors. The resistors *R*_1_, *R*_2_, and *R_f_* were implemented off-chip to make the architecture adjustable. A negative feedback loop is created around the control amplifier, which provides a virtual short at the RE. The transfer function of the proposed potentiostat is expressed by:

(5) $$V_{\mathrm{out}}=I_{F}\cdot R_{f}\cdot \left(1+\frac{R_{2}}{R_{1}}\right)+V_{\mathrm{cm}}$$

where *I_F_* is the sensor current.

The control block consists of a control amplifier and resistor *R_f_*. The control block supplies the desired cell potential between the WE and the RE in order to maintain the function of the amperometric sensor. The WE is connected to voltage *V_WE_*, taken from the supply voltage of the potentiostat, to prevent it from picking up environmental noise and interference. In order to prevent current from flowing into the RE, which affects the cell potential, the voltage of the RE is forced through the virtual short of the control amplifier. Thus, the cell potential is given by:

(6) $$V_{\mathrm{cell}}=V_{\mathrm{WE}}-V_{\mathrm{RE}}=V_{\mathrm{WE}}-V_{\mathrm{bias}}$$

**Figure 5.**
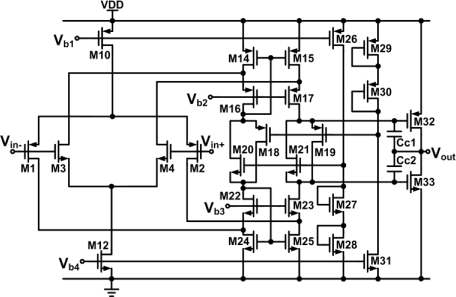
Schematic of the control amplifier.

From Equation (6), the ICMR of the control amplifier restricts the voltage swing of *V_cell_*. Therefore, the control amplifier must have a rail-to-rail ICMR to provide the maximum swing of *V_cell_* for various sensor applications. The voltage gain of the control amplifier must also be considered because it defines the accuracy of the virtual short voltage. It is usually suggested that the gain of the control amplifier be larger than 80 dB [27]. Moreover, the number of stages of the architecture of the control amplifier should be as few as possible to increase stability. From the above requirements, a rail-to-rail input/output operational amplifier with a folded cascode input stage is adopted as the control amplifier to obtain the minimum number of poles, sufficient gain, and rail-to-rail input and output. A schematic of the control amplifier is shown in Figure 5. The transistor dimensions for the control amplifier of Figure 5 are listed in Table 2. The bias circuit of this amplifier is the same as that of the DDA.

The amplifier block includes a negative feedback DDA which is used to linearly amplify the converted voltage signal across R_f_. The architecture of the DDA of the amplifier block is the same as that in Figure 3.

### 2.2. Microcontroller Unit

**Figure 6.**
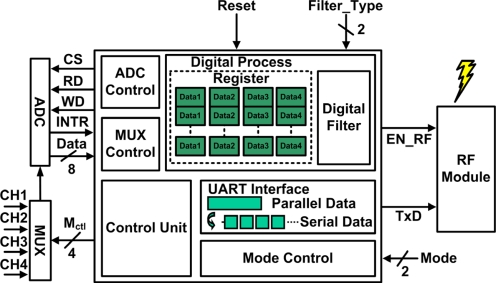
Block diagram of the proposed MCU.

A microcontroller unit is programmed to filter unwanted noise signals, control peripheral circuits to reduce system power consumption, and transmit the data via a universal asynchronous receiver transmitter (UART) interface. The proposed microcontroller unit is implemented on an Altera MAX II EPM2210F324 FPGA device. This FPGA device is adopted due to its compact size and low cost; however, the number of available logic elements is limited to 2,210. Figure 6 shows a block diagram of the proposed MCU. The blocks of the proposed MCU are described in detail below.

#### 2.2.1. MUX/ADC Control

In order to reduce hardware for multi-channel applications, a CMOS switch (4066BP, TOSHIBA), is used to as a MUX. Four-channel control is provided by the proposed MCU, allowing four types of analyte to be simultaneously detected. The sample frequency of each channel is set to 24 Hz due to the long response time of the adopted electrochemical sensors (several tens of seconds). An ADC with 8-bit resolution (ADC0804, National Semiconductor) is adopted. The clock frequency is set to 192 kHz, which is supplied by the MCU.

#### 2.2.2. Digital Filter

**Figure 7.**
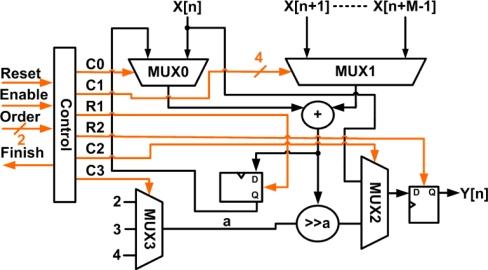
Structure of the moving average filter.

**Figure 8.**
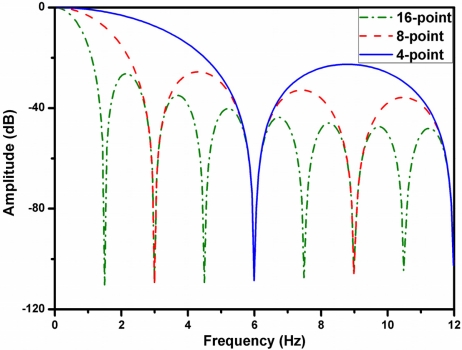
Simulated frequency response of the adjustable cascade moving average filter.

In order to reduce interference and noise from the sensors and the power line, a low-pass filter (LPF) is used. A moving average filter, which is a sample low-pass finite impulse response (FIR) filter, has the properties of low hardware requirements and low computation time [28], and is thus adopted here to satisfy the limitations of available hardware in the FPGA device. In order to conform to the response times of various sensors, the architecture of adjustable points is utilized to provide three types of 4-point, 8-point, and 16-point in the average. The types are switched by Filter_Type. The equation of the moving average filter can be expressed by:

(7) $$y\left[n\right]=\frac{1}{M}\sum_{j=0}^{M-1}x\left[n+j\right]$$

where *y*[*n*] is the output signal, *x*[*n* + *j*] is the input signal, and M is the number of points in the average. Figure 7 shows the structure of the moving average filter. In this work, two identical moving average filters are cascaded to obtain faster roll-off. The simulated frequency response of the adjustable cascade moving average filter is shown in Figure 8.

#### 2.2.3. UART Interface Communication

The UART interface is widely used for serial communication. The transport protocol of the UART is simpler than that of USB. The relatively slow data transmission of UART is sufficient for this work due to the long response time of the sensors. Thus, a UART interface, which is provided by the adopted RF transceiver module, is adopted to convert parallel data into serial data for the transmitter. The baud rate is set to 1,200 bps.

#### 2.2.4. Mode Control

**Figure 9.**
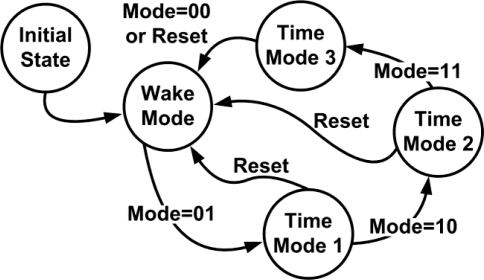
State diagram of the mode control.

The signals of the electrochemical sensors need not be detected continuously due to the long response time of the sensors. Thus, the function of mode control is implemented in the proposed MCU to reduce the power consumption of the system. The modes can be changed by a trigger signal, which is generated by the push-button switch on the FPGA board. Figure 9 shows the state diagram of the mode control. After turning on the power, the hardware is properly configured and automatically switched from Initial State to Wake Mode. In Wake Mode, the system is always enabled. In Time Mode 1, the system alternates between wake (1 s) and sleep (1 s) modes. In Time Mode 2, the system alternates between wake (1 s) and sleep (5 s) modes. In Time Mode 3, the system alternates between wake (1 s) and sleep (10 s) modes.

#### 2.2.5. Control Unit

**Figure 10.**
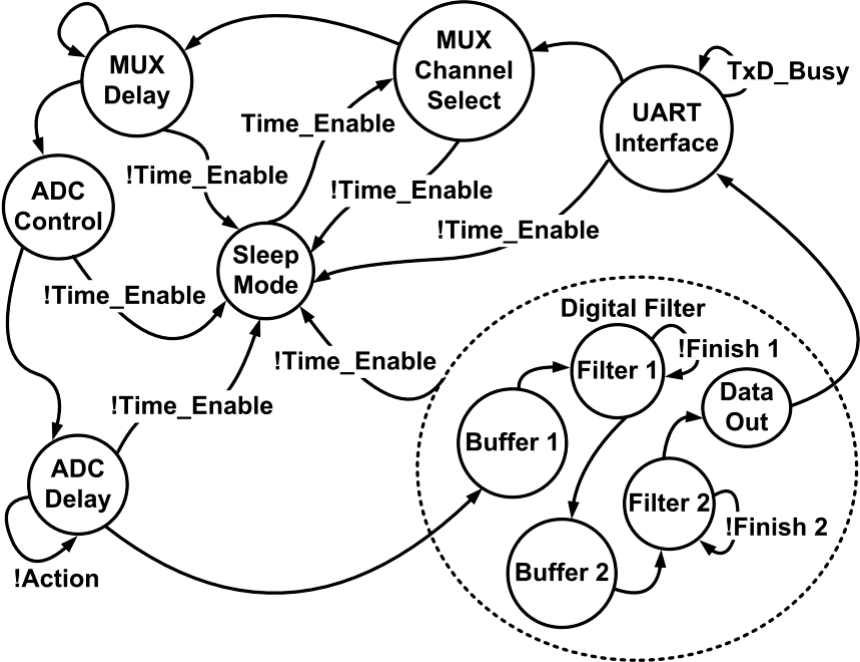
State diagram of the control unit.

The peripheral circuits and each function block of the MCU need a central control unit to manage the proposed system and maintain correct operation. The state diagram of the control unit is shown in Figure 10. Time_Enable is generated to wake up the system. When the system is disabled, it enters sleep mode. The duration of sleep mode depends on the set operation mode.

### 2.3. RF Transceiver and GUI

An RF transceiver module (APC220-43, APPCON Technologies) is adopted to transmit data wirelessly for increased mobility. This highly integrated semi-duplex low-power transceiver module provides high-efficiency error correction to ensure correct data transmission in the presence of strong interference. The communication distance is up to 1,000 m. The module is small and low-cost, and supports the UART interface. The operation frequency of the module is set to 433 MHz, which is an industrial scientific medical (ISM) frequency band. Gaussian frequency-shift keying (GFSK) modulation is used. The GUI was developed in Visual Basic to provide a user-friendly interface for users to display data in real-time and automatically upload the data to a database over the internet. The GUI can simultaneously display four channels. The database runs on a Microsoft structured query language (SQL) server.

## 3. Measurement Results

### 3.1. Readout Circuits

**Figure 11.**
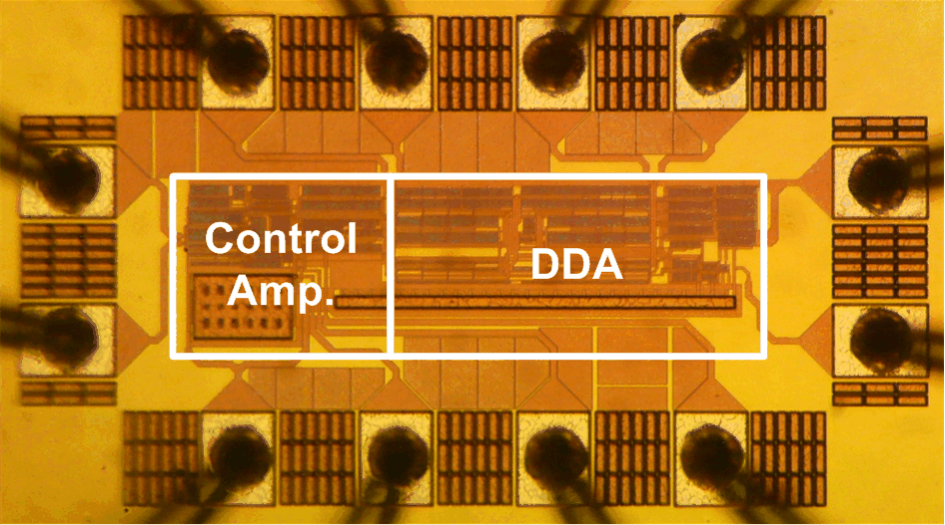
Micrograph of the fabricated potentiostat.

The proposed readout chip was implemented in the TSMC 0.18-μm CMOS technology. A micrograph of the fabricated potentiostat is shown in Figure 11. The DDA part of the potentiostat is used only to measure the open-circuit potential for potentiometric electrochemical sensors. The total core area of the chip is 0.05 mm^2^. The total power consumption of the control amplifier and the DDA is 82 μW at a 1.8 V supply voltage. Peripheral devices are supplied with 3.3 V. In order to use a single power supply for the whole system, a low-dropout high-output-accuracy CMOS voltage regulator (VRH1802LTX, Analog Semiconductor) is used to convert 3.3 V to 1.8 V to supply the implemented readout chip.

**Figure 12.**
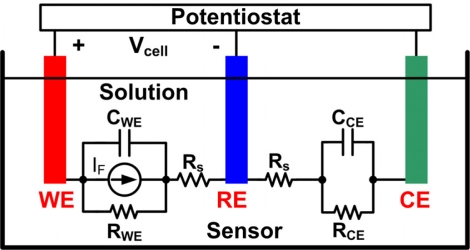
Equivalent model of the three-electrode amperometric sensor.

To evaluate the performance of the proposed potentiostat, the equivalent model of the electrochemical sensor, shown in Figure 12, was built to serve as a platform for data measurements. *C_WE_* and *C_CE_*, chosen according to [24], are set to 1 uF and 1 nF, respectively. *R_s_* is 10 Ω and *R_CE_* is 1 kΩ. A varying voltage signal, *V_in_*, was applied to the WE and the RE was biased at a constant voltage, *V_bias_*, by the virtual short of the input terminals of the control amplifier. The value of *R_WE_* depends on the desired current. Therefore, the emulated sensor current, *I_F_*, is generated by:

(8) $$I_{F}=\frac{V_{\mathrm{in}}-V_{\mathrm{bias}}}{R_{\mathrm{WE}}}$$

**Figure 13.**
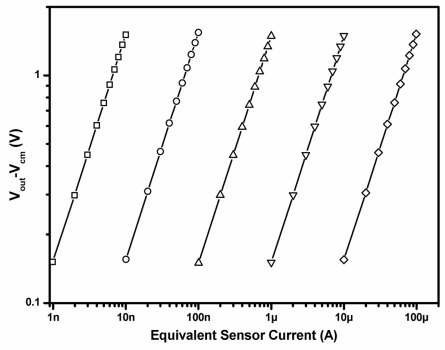
Equivalent sensor current *versus V_out_* − *V_cm_* of the proposed potentiostat. The R^2^ value of each current range is greater than 0.99998.

An important specification of a readout circuit is its linearity. The coefficient of determination (R^2^) for a linear regression was used to analyze the linearity of the proposed potentiostat. During linearity measurement, a DC sweep voltage signal was applied to convert the equivalent current signal. For each current range measurement, feedback resistor *R_f_* was changed to maintain the voltage variation across *R_f_* of 10 mV to 100 mV. The closed-loop gain of the DDA was set to 15 V/V. Figure 13 shows the experimental results of the relation between the equivalent sensor current and the absolute variation of the voltage at the output terminal. The measurement results show that the R^2^ value is greater than 0.99998 in each measured current range, indicating that the proposed potentiostat has high linearity and a wide detectable current range. Additionally, the control amplifier and the DDA were experimentally verified; both have a rail-to-rail ICMR and a rail-to-rail output swing, making them suitable for multiple-sensor applications. The dynamic range of *V_out_* is about 65 dB and the voltage swing of the input common-mode voltage is 1.79 V. Moreover, the proposed potentiostat has a small number of components, reducing component noise, and high values of CMRR and power supply rejection ratio (PSRR) to suppress common-mode and power supply noise, respectively. It was verified that the CMRR and the PSRR of the proposed DDA are both greater than 90 dB for frequencies below 100 Hz. The input-referred voltage noise of the proposed DDA, integrated from 0.1 to 100 Hz, was also measured to be 2.8 μVrms.

The experimental results of the proposed potentiostat are summarized in Table 3. In this work, the WE was connected to a bias voltage due to its insensitivity to environment noise and interference. Compared to [29], the proposed potentiostat has a relatively simple architecture that can supply a rail-to-rail value of V_cell_ for various sensor applications. Furthermore, the proposed potentiostat avoids the mismatched current issue of [24] and [25]. The proposed architecture achieved excellent linearity with an R^2^ value of 0.99998 and a wide detectable current range of 1 nA to 100 μA.

### 3.2. Proposed Telemetry System

**Figure 14.**
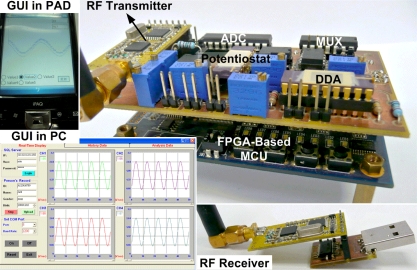
Photograph of the proposed system.

The proposed telemetry system for portable applications is shown in Figure 14. The PCBs are integrated in a stacked manner for improved portability. The board size of the transmitter unit is 5.6 cm × 8.7 cm and the height is 3 cm. In operation mode, the total power consumption of the transmitter unit is 157.25 mW with a supply voltage of 3.3 V. In sleep mode, the transmitter unit consumes 87.83 mW. Table 4 shows the overall power utilization for the proposed telemetry system. Because USB is the most widely used computer interface, the receiver unit receives UART formatted data transmitted via a USB port on a UART/USB interface IC (FT232RL, FTDI Chip).

**Figure 15.**
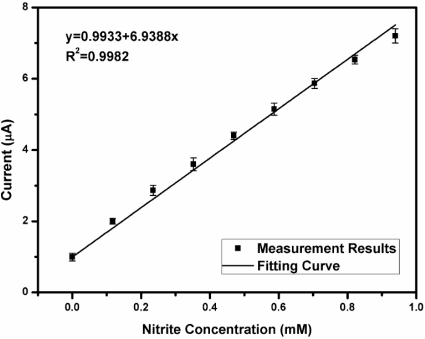
Nitrite concentration *versus* the detected current signals.

**Figure 16.**
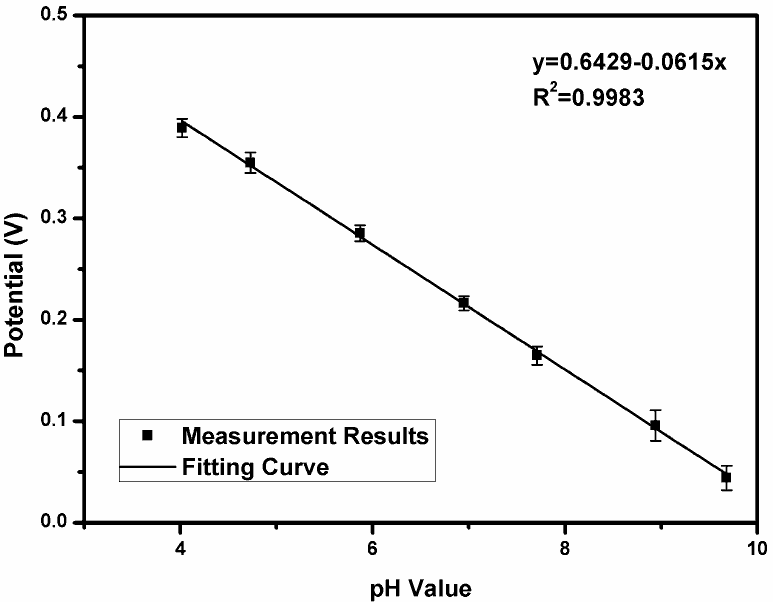
pH value *versus* the detected potential signals.

Electrochemical analysis was conducted by integrating the proposed system with a nitrite amperometric sensor [30] and a pH potentiometric sensor [31] *in vitro*. The nitrite sensor was immersed into a 0.1 M phosphate-buffered-saline (PBS) solution (pH 6). The concentration of nitrite was increased by 0.12 mM per step. A V_cell_ of 0.7 V *vs*. Ag/AgCl was applied. Figure 15 shows the detected current signals *versus* nitrite concentration. Linearity with an R^2^ value of 0.9982 was achieved in this electrochemical analysis. For the potentiometric sensor measurement, the pH sensor was immersed into aqueous solution. The pH value of the aqueous solution was increased and the potential *vs*. Ag/AgCl was measured. Figure 16 shows the detected signals for various pH values. An R^2^ value of 0.9983 was obtained during the pH measurement. The measurement results are represented by using mean ±S.D. with five observations and show that the proposed system has high linearity because an R^2^ value greater than 0.99 was obtained in each experiment. The experimental results of the proposed system are summarized in Table 5.

## 4. Conclusions

A real-time telemetry system with high-linearity readout circuits for electrochemical sensors and a user-friendly GUI were developed for conveniently detecting analytes in real-time and uploading the data to a database over the Internet. Remote users can easily obtain the detected data over the Internet from the database. The proposed readout circuits were fabricated in the TSMC 0.18-μm CMOS technology. An FPGA-based MCU was implemented to filter unwanted noise and to manage the system power consumption. Electrochemical experiments were conducted to verify the feasibility of integrating the proposed system with an amperometric nitrite sensor and a potentiometric pH sensor. The proposed system has high linearity (an R^2^ value greater than 0.99), a small size of 5.6 cm × 8.7 cm, and high integration.

## Figures and Tables

**Table 1.** Transistor dimensions of the DDA.

**Devices**	**W/L (μm)**	**Devices**	**W/L (μm)**
MN(P)1, MN(P)2	16.6/20	MN(P)3, MN(P)4	3/20
MN(P)5, MN(P)7	3/20	MN(P)6, MN(P)8	16.6/20
MN(P)9	3/4	MN(P)10	112/10
MN(P)11, M26	14/10	MN(P)12, Mb3	16/10
MN(P)13, M31	2/10	M14–M17	98/4
M18–M21	10/5	M22–M25	14/4
M27, M28	3/8	M29, M30	11.5/4
M32	138.5/2	M33	18/2
Mb1, Mb2	4/10	Mb4, Mb6, Mb7	4/4
Mb5	2/16	Mb8	12/16
Mb9–Mb11	16/4	Mb12–Mb14	28/10
	

**Table 2.** Transistor dimensions of the control amplifier.

**Devices**	**W/L (μm)**	**Devices**	**W/L (μm)**
M1, M2	19/20	M3, M4	3.4/20
M10	28/10	M12	4/10
M14, M15	24/4	M16, M17	12/4
M18–M21	6/20	M22, M23, M27, M28	2/4
M24, M25	4/4	M26	14/10
M29, M30	11.6/4	M31	2/10
M32	139.8/2	M33	24/2
	

**Table 3.** Specification comparison of the proposed potentiostat.

**Specification**	**[24]**	**[25]**	**[29]**	**This Work**
**Power Supply (V)**	1.8	1.8	±0.9	1.8
**Process (μm)**	0.18	0.18	0.18	0.18
**I_range_ (A)**	1 n–1 μ	1 n–200 n	-	1 n–100 μ
**Linearity (R^2^)**	0.9984	-	0.98	0.99998
**I_readout_ Electrode**	CE	CE	WE	CE
**Supply RtR *V_cell_***	No	No	Yes	Yes
**Architecture**	SE	SE	FD	SE
**Output Signal**	Freq.	Freq.	V_CT_	V_CT_
**Core Area (mm^2^)**	0.02	0.04	0.45	0.05
**Power (μW)**	70	171	15,840	82

FD: Fully-differential; SE: Single-ended; CT: Continuous time; RtR: Rail-to-Rail.

**Table 4.** Power consumption of each block.

**Block**	**Power Consumption**	
	**Wake Mode**	**Sleep Mode**
**Readout Circuit**	0.14 mW	
**ADC**	3.18 mW	
**MCU**	66.9 mW	
**RF Module**	69.43 mW	0.01 mW
**Bias Circuit & Regulator**	17.6 mW	

**Table 5.** Experimental results of the proposed system.

	
	**Specifications**
**System Supply Voltage**	3.3 V
**Detectable Current Range of Potentiostat**	1 nA–100 μA
**CMRR of IA**	>90 dB
**Input Referred Noise of IA**	2.8 μVrms
**Linearity (R^2^)**	>0.99
**Power Consumption (Wake Mode)**	157.25 mW
**Power Consumption (Sleep Mode)**	87.83 mW
**Operation Frequency of RF Module**	433 MHz
**Used Logic Elements of MCU**	1.8 k
**Transmitter Unit Size**	5.6 cm × 8.7 cm
**Receiver Unit Size**	4 cm × 1.8 cm
